# Supplementary material for: Electrocatalytic CO2 Reduction and H2 Evolution by a Copper (II) Complex with Redox-Active Ligand
Source: Molecules. 2022 Feb 18;27(4):1399. doi: 10.3390/molecules27041399 (PMC8874443; doi:10.3390/molecules27041399)

checkCIF/PLATON report

Structure factors have been supplied for datablock(s) mo_20180625-zghy-6_0m_sq_s

THIS REPORT IS FOR GUIDANCE ONLY. IF USED AS PART OF A REVIEW PROCEDURE FOR PUBLICATION, IT SHOULD NOT REPLACE THE EXPERTISE OF AN EXPERIENCED CRYSTALLOGRAPHIC REFEREE.

No syntax errors found. CIF dictionary Interpreting this report

Datablock: mo_20180625-zghy-6_0m_sq_s

```
Bond precision:  C-C = 0.0125 A          Wavelength=0.71073

Cell:            a=27.32(2)     b=13.544(13)       c=21.773(19)
                 alpha=90       beta=121.34(2)     gamma=90
Temperature:     173 K
```

	Calculated	Reported
Volume	6881(10)	6881(11)
Space group	C 2/c	C 1 2/c 1
Hall group	-C 2yc	-C 2yc
Moiety formula	C26 H18 Cu N9 O9 [+ solvent]	C26 H18 Cu N9 O9
Sum formula	C26 H18 Cu N9 O9 [+ solvent]	C26 H18 Cu N9 O9
Mr	664.04	664.03
Dx,g cm-3	1.282	1.282
Z	8	8
Mu (mm-1)	0.693	0.693
F000	2704.0	2704.0
F000’	2707.75	
h,k,lmax	33,16,26	33,16,26
Nref	6413	6343
Tmin,Tmax	0.905,0.946	0.658,0.746
Tmin’	0.877	

```
Correction method= # Reported T Limits: Tmin=0.658 Tmax=0.746
AbsCorr = MULTI-SCAN

Data completeness= 0.989          Theta(max)= 25.495

R(reflections)= 0.0908( 3448)     wR2(reflections)= 0.3016( 6343)

S = 1.040                   Npar= 408
```

The following ALERTS were generated. Each ALERT has the format
test-name_ALERT_alert-type_alert-level.
Click on the hyperlinks for more details of the test.

Alert level B

```
PLAT232_ALERT_2_B Hirshfeld Test Diff (M-X)  Cu1      --O4       .       34.5 s.u.
PLAT241_ALERT_2_B High   'MainMol' Ueq as Compared to Neighbors of         O4 Check
PLAT430_ALERT_2_B Short Inter D...A Contact  O1       ..O8       .       2.76 Ang.
                                              1-x,1-y,1-z  =      5_666 Check
PLAT430_ALERT_2_B Short Inter D...A Contact  O8       ..N1       .       2.82 Ang.
                                              1-x,1-y,1-z  =      5_666 Check
```

Alert level C

```
PLAT084_ALERT_3_C High wR2 Value (i.e. > 0.25) ...................       0.30 Report
PLAT220_ALERT_2_C Non-Solvent  Resd 1  N   Ueq(max)/Ueq(min) Range        3.3 Ratio
PLAT220_ALERT_2_C Non-Solvent  Resd 1  O   Ueq(max)/Ueq(min) Range        3.6 Ratio
PLAT234_ALERT_4_C Large Hirshfeld Difference N8       --C12      .       0.19 Ang.
PLAT241_ALERT_2_C High   'MainMol' Ueq as Compared to Neighbors of         O3 Check
PLAT242_ALERT_2_C Low    'MainMol' Ueq as Compared to Neighbors of        Cu1 Check
PLAT242_ALERT_2_C Low    'MainMol' Ueq as Compared to Neighbors of         N1 Check
PLAT242_ALERT_2_C Low    'MainMol' Ueq as Compared to Neighbors of         N8 Check
PLAT242_ALERT_2_C Low    'MainMol' Ueq as Compared to Neighbors of         N9 Check
PLAT242_ALERT_2_C Low    'MainMol' Ueq as Compared to Neighbors of        C12 Check
PLAT260_ALERT_2_C Large Average Ueq of Residue Including       Cu1      0.102 Check
PLAT341_ALERT_3_C Low Bond Precision on  C-C Bonds ...............    0.01255 Ang.
```

Alert level G

```
PLAT003_ALERT_2_G Number of Uiso or Uij Restrained non-H Atoms ...         45 Report
PLAT012_ALERT_1_G No       _shelx_res_checksum Found in CIF ......     Please Check
PLAT014_ALERT_1_G No       _shelx_fab_checksum Found in CIF ......     Please Check
PLAT072_ALERT_2_G SHELXL First  Parameter in WGHT  Unusually Large       0.18 Report
PLAT128_ALERT_4_G Alternate Setting for Input Space Group     C2/c       I2/a Note
PLAT232_ALERT_2_G Hirshfeld Test Diff (M-X)  Cu1      --O3       .        8.5 s.u.
PLAT606_ALERT_4_G VERY LARGE Solvent Accessible VOID(S) in Structure        ! Info
PLAT794_ALERT_5_G Tentative Bond Valency for Cu1       (II)      .       2.25 Info
PLAT860_ALERT_3_G Number of Least-Squares Restraints .............        408 Note
PLAT869_ALERT_4_G ALERTS Related to the Use of SQUEEZE Suppressed           ! Info
```

---

0 **ALERT level A** = Most likely a serious problem - resolve or explain
4 **ALERT level B** = A potentially serious problem, consider carefully
12 **ALERT level C** = Check. Ensure it is not caused by an omission or oversight
10 **ALERT level G** = General information/check it is not something unexpected

2 ALERT type 1 CIF construction/syntax error, inconsistent or missing data
16 ALERT type 2 Indicator that the structure model may be wrong or deficient
3 ALERT type 3 Indicator that the structure quality may be low
4 ALERT type 4 Improvement, methodology, query or suggestion
1 ALERT type 5 Informative message, check

It is advisable to attempt to resolve as many as possible of the alerts in all categories. Often the minor alerts point to easily fixed oversights, errors and omissions in your CIF or refinement strategy, so attention to these fine details can be worthwhile. In order to resolve some of the more serious problems it may be necessary to carry out additional measurements or structure refinements. However, the purpose of your study may justify the reported deviations and the more serious of these should normally be commented upon in the discussion or experimental section of a paper or in the "special_details" fields of the CIF. checkCIF was carefully designed to identify outliers and unusual parameters, but every test has its limitations and alerts that are not important in a particular case may appear. Conversely, the absence of alerts does not guarantee there are no aspects of the results needing attention. It is up to the individual to critically assess their own results and, if necessary, seek expert advice.

Publication of your CIF in IUCr journals

A basic structural check has been run on your CIF. These basic checks will be run on all CIFs submitted for publication in IUCr journals (*Acta Crystallographica*, *Journal of Applied Crystallography*, *Journal of Synchrotron Radiation*); however, if you intend to submit to *Acta Crystallographica Section C* or *E* or *IUCrData*, you should make sure that full publication checks are run on the final version of your CIF prior to submission.

Publication of your CIF in other journals

Please refer to the *Notes for Authors* of the relevant journal for any special instructions relating to CIF submission.

PLATON version of 18/02/2019; check.def file version of 18/02/2019

Datablock mo_20180625-zghy-6_0m_sq_s - ellipsoid plot

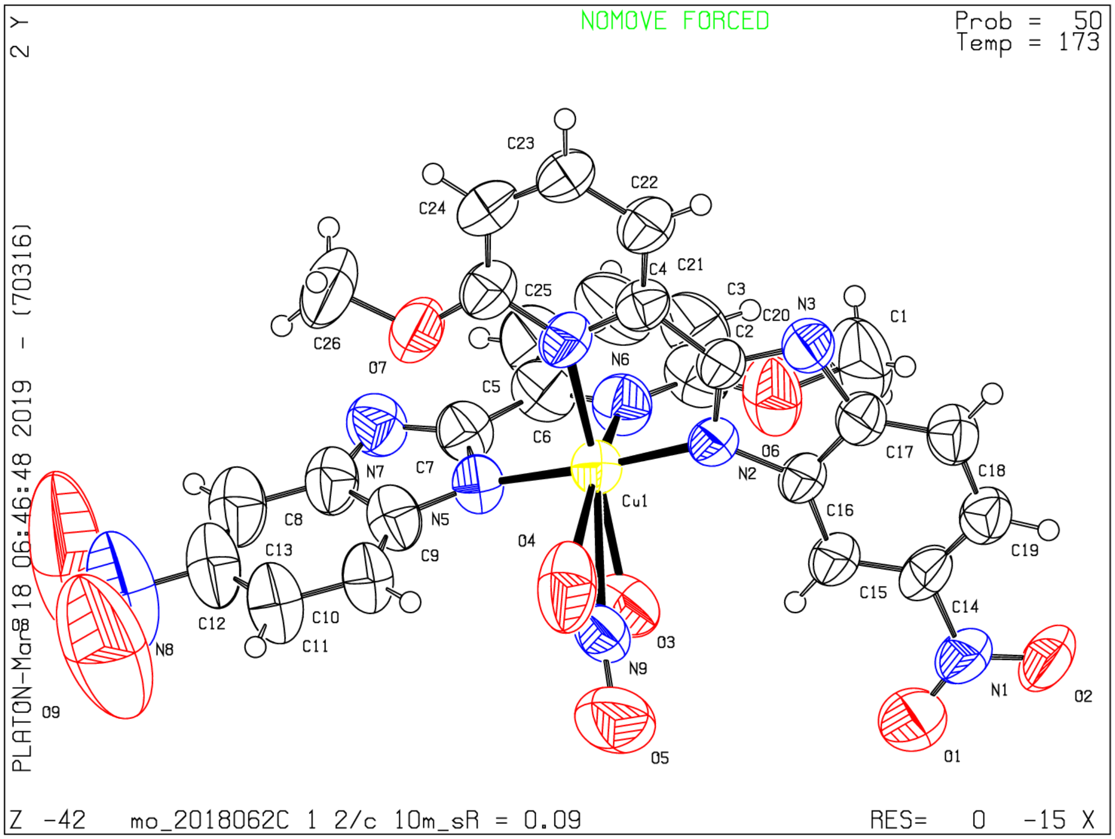

Supplement: Supplementary file 1 [file molecules-27-01399-s001.zip › complex1checkcif.pdf]
