# Supplementary material for: Electrocatalytic CO2 Reduction and H2 Evolution by a Copper (II) Complex with Redox-Active Ligand
Source: Molecules. 2022 Feb 18;27(4):1399. doi: 10.3390/molecules27041399 (PMC8874443; doi:10.3390/molecules27041399)
Supplement: Supplementary file 1 [file molecules-27-01399-s001.zip › Supplementary Figures and Tables.pdf]

SUPPORTING INFORMATION:

Electrocatalytic CO_2 reduction and H_2 evolution by a copper (II) complex with redox-active ligand

Jingjing Li [1], Shifu Zhang [1], Jinmiao Wang [1], Xiaomeng Yin [1], Zhenxing Han [1], Guobo Chen [1], Dongmei Zhang [1] and Mei wang [1,2*]

Contents **Page No**

Table S1. Crystallological data for the complex 1 2

Table S2. The main key length of the complex 1.......... 2

Table S3. The main key angle of the complex 1 3

Table S4. Cartesian coordinates for 1 3

Figure S1. IR spectrum of the complex 1.. .... 5s

Figure S2. HOMO-LUMO orbitals of complex 1.......... 6

Figure S3. (a) LUMO+1 orbital; (b) LUMO+2 orbital; (c) HOMO-1 orbital; (d) HOMO-2 orbital.......... 6

Figure S4. Powder X-ray diffraction (PXRD) patterns of complex 1 7

Figure S5. Cyclic voltammetry of 2 mM ligand L^1 under 1 atm Ar at scan rate 100 mV s^{-1}.......... 7

Table S5. The reduction potentials and peak currents of complex 1 under 1 atm Ar in a 0.1 M $^nBu_4NPF_6$ CH_3CN supporting electrolyte. 7

Table S6. The reduction potentials and peak currents of complex 1 under 1 atm CO_2 in a 0.1 M $^nBu_4NPF_6$ CH_3CN supporting electrolyte. 8

Table S7. The reduction potentials and peak currents of complex 1 containing different concentrations of H_2O under CO_2. 8

Table S8. The reduction potentials and peak currents of complex 1 with different concentrations under 1 atm CO_2 in a 0.1 M $^nBu_4NPF_6$ CH_3CN supporting electrolyte.......... 8

Figure S6. (a) CPE for H_2 evolution (black line) at -1.97 V vs NHE on GCE (0.07 cm^2); (b) CPE for CO_2 reduction (red line) at -1.15 V vs NHE on GCE (0.07 cm^2) at the same condition as using FTO. 9

Figure S7. CPE with 2 mM complex 1 (rose red line), rinse test (green line) and the blank experiment without 1 (blue line) on an FTO working electrode (1.0 cm^2).......... 10

Figure S8. The in-situ UV-Vis spectroelectrochemistry of complex 1 in CO_2 atmosphere.......... 10

Figure S9. (a) The amount of material in the proton supply system that is combined with the electrocatalytic reduction CO_2 product. (b) The Faraday efficiency curves of the electrocatalytic reduction products CO and H_2 in the proton supply H_2O system. 11

Figure S10. Cyclic voltammetry of complex 1 in the presence (green) and absence (black) of CO_2 recorded at 100 mV s^{-1} at glassy carbon in a 0.1 M $^nBu_4NPF_6$ CH_3CN supporting electrolyte.......... 12

Figure S11. Cyclic voltammograms of complex 1 (2 mM) recorded in the presence of 0.58 mM TsOH·H_2O under 1 atm Ar at scan rate range from 100 to 500 mV s^{-1} in CH_3CN (0.1 M $^nBu_4NBF_6$) at a glassy carbon electrode. 13

Figure S12. The *in-situ* UV-Vis spectroelectrochemistry of complex 1 in Ar atmosphere. 13

Figure S13. Cyclic voltammograms of complex 1 (2 mM, red trace) and in the presence of 10 mM TsOH·H_2O (black trace) in CH_3CN (0.1 M $^nBu_4NPF_6$) at a glassy carbon electrode and 100 mV s^{-1}.......... 13

Figure S14. Cyclic voltammograms recorded in the absence (black trace) or in the presence of 10 mM of TsOH·H_2O (red trace). Scan rate: 100 mV s^{-1}. Working electrode: glassy carbon. Counter electrode: Pt wire. Reference

electrode: Ag/AgCl. 14
Figure S15. DLS spectra of the electrolyte before and after the CPE test. (a) DLS of the complex 1 containing 0.58mM TsOH·H_2O in CH_3CN (0.1 M $^nBu_4NPF_6$) before and after 4000 s electrolysis; (b) DLS of the complex 1 in CH_3CN (0.1 M $^nBu_4NPF_6$) under 1atm CO_2 before and after 4000 s electrolysis. 15
Figure S16. The Faraday efficiency curves of the electrocatalytic hydrogen evolution products H_2 in the proton supply 0.58 mM TsOH·H_2O system. 16
Figure S17. Plot of the magnetic moments (μ_{eff}) versus the temperature T for solid sample of complex 1. 17
Table S9. The molecular geometry of complex 1 as predicted by SHAPE 2.1. 17

Table S1. Crystallological data for the complex **1**

Parameter	**1**
Empirical formula	$C_{26}H_{18}CuN_{10}O_{12}$
Formula weight	726.03
Temperature (K)	173
Wavelength (Å)	0.71073
Crystal system	Monoclinic
Space group	*C12 / c1*
a (Å)	27.32(2)
b (Å)	13.544(13)
c (Å)	21.773(19)
α (˚)	90
β (˚)	121.34(2)
γ (˚)	90
Volume (Å^3)	6881(11)
Z	8
Calculateddensity(g/cm^3)	1.282
Absorption coefficient (mm^{-1})	0.693
F (000)	2704
Crystal size (mm)	0.19 × 0.12 × 0.08
ϑ range for data collection (˚)	1.738 to 25.495
Limiting indices	$-32 \le h \le 33$
	$-16 \le k \le 16$
	$-26 \le l \le 24$
Reflections collected / unique	24001/6343
	[R_{int} = 0.0700]
Completeness to *ϑ* = 25.495	0.989
Max. and min. transmission	0.7455 and 0.6580
Data / restraints / parameters	6343 / 408 / 408
Goodness of fit on F^2	1.040
R_1^a, wR_2^b [$I > 2\sigma(I)$]	R_1 = 0.0908
	wR_2 = 0.2737
R_1^a, wR_2^b (all data)	R_1 = 0.1447
	wR_2 = 0.3016
Largest diff. peak and hole (e. Å^{-3})	0.951 and -0.555

Table S2. The main key length of the complex 1

bond	d. Å	bond	d. Å
Cu(1)-N(2)	1.9419	Cu(1)-N(6)	2.2273
Cu(1)-N(4)	2.2248	Cu(1)-O(3)	2.0792
Cu(1)-N(5)	1.9525	Cu(1)-O(4)	2.5717

Table S3. The main key angle of the complex 1

Angle	ω, deg	Angle	ω, deg
N(2)-Cu(1)-N(5)	176.025	O(4)-Cu(1)-N(6)	161.059
N(2)-Cu(1)-N(4)	78.854	O(4)-Cu(1)-O(3)	52.789
N(2)-Cu(1)-N(6)	98.572	O(4)-Cu(1)-N(4)	100.994
N(5)-Cu(1)-N(4)	97.976	O(3)-Cu(1)-N(2)	91.4
N(5)-Cu(1)-N(6)	79.378	O(3)-Cu(1)-N(2)	91.471
N(4)-Cu(1)-N(6)	97.506	O(3)-Cu(1)-N(4)	152.574
O(4)-Cu(1)-N(5)	93.984	O(3)-Cu(1)-N(6)	109.36
O(4)-Cu(1)-N(2)	89.003	O(3)-Cu(1)-N(5)	92.424

Table S4. Cartesian coordinates for 1

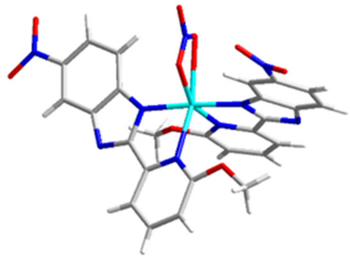

Coordinates (Angstroms)			
Symbol	X	Y	Z
Cu	10.5242	7.5612	11.1395
O	11.4512	1.7607	12.1601
O	13.5208	1.1038	12.2698
O	9.4717	5.8131	11.539
O	9.0756	7.0551	13.2033
O	8.2652	5.0424	13.1029
O	13.0844	6.1408	9.4915
O	9.9505	10.9124	11.022
O	3.8773	11.4894	9.0973
O	3.4229	10.5901	11.1894
N	12.6422	1.9815	12.2679
N	12.0746	6.9088	12.1099
N	14.0874	7.2447	12.9634
N	11.4834	9.4727	11.7528
N	9.0431	8.3174	10.1163

N	11.359	7.4655	9.0768
N	8.4829	9.5553	8.3534
N	4.1293	10.7485	10.1684
N	8.9744	6.0325	12.6473
C	14.3797	5.5422	9.1456
H	14.2384	4.7824	8.5431
H	14.8251	5.2334	9.962
H	14.9427	6.2113	8.7012
C	12.4359	6.898	8.5915
C	12.8513	7.1025	7.2544
H	13.6159	6.6555	6.9141
C	12.143	7.9449	6.4659
H	12.4588	8.1724	5.5975
C	10.9686	8.4596	6.9364
H	10.3977	8.9661	6.3711
C	10.6284	8.2212	8.2846
C	9.3865	8.7332	8.9243
C	7.4688	9.6257	9.2739
C	7.8106	8.8645	10.386
C	6.9591	8.7359	11.4478
H	7.2017	8.232	12.2159
C	5.7053	9.3779	11.3623
H	5.0767	9.2709	12.0671
C	5.3768	10.1485	10.2912
C	6.188	10.284	9.2237
H	5.9222	10.7932	8.4669
C	13.0707	3.363	12.4929
C	12.2098	4.3612	12.1526
H	11.3453	4.1865	11.7956

C	12.6817	5.6573	12.3646
C	13.9267	5.87	12.9132
C	14.7688	4.842	13.2666
H	15.6184	5.0045	13.6571
C	14.3064	3.5323	13.0211
H	14.8495	2.7806	13.2238
C	12.9475	7.8027	12.4799
C	12.6811	9.2208	12.3163
C	13.5329	10.2068	12.7793
H	14.3423	9.9955	13.2312
C	13.1488	11.5259	12.5487
H	13.7179	12.2384	12.8165
C	11.9662	11.7995	11.9425
H	11.7134	12.7002	11.7789
C	11.1404	10.777	11.5669
C	9.415	12.2505	10.8918
H	9.9985	12.7788	10.306
H	9.3716	12.6718	11.7751
H	8.5168	12.2059	10.505

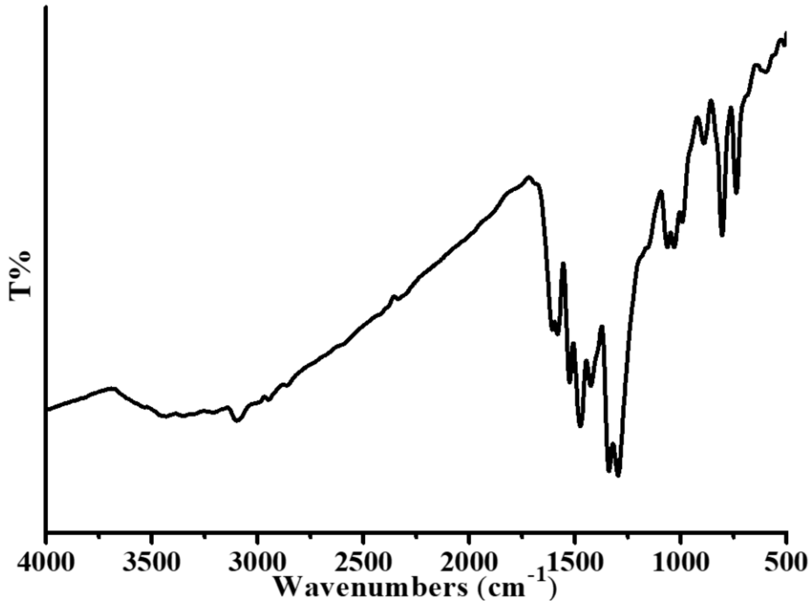

Figure S1. IR spectrum of the complex **1**.

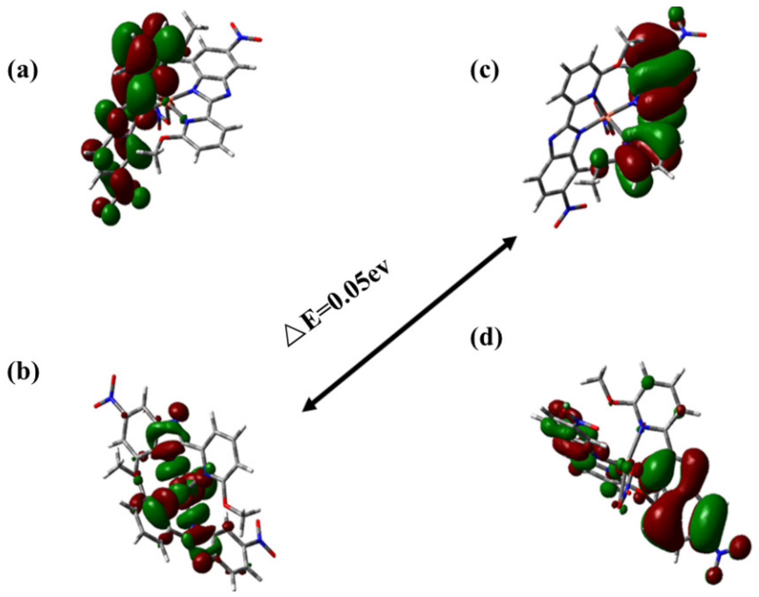

Figure S2. HOMO-LUMO orbitals of complex **1**.

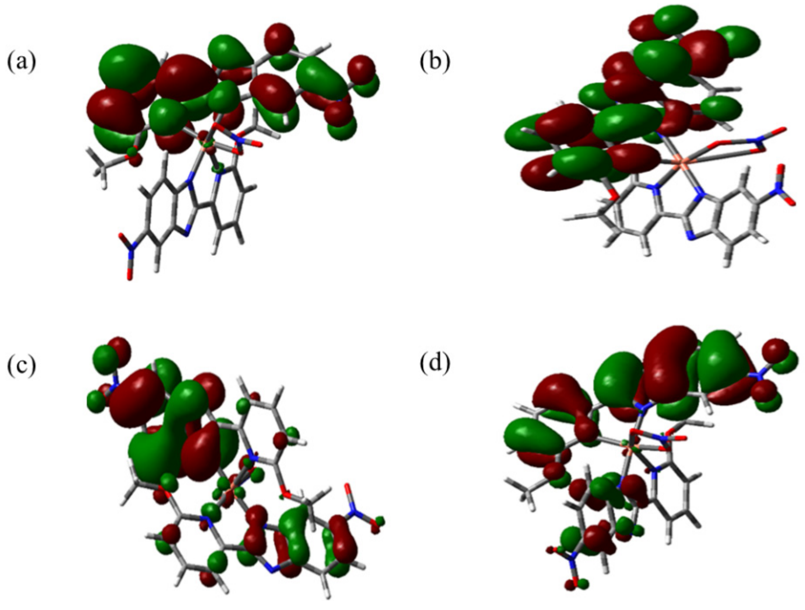

Figure S3. (**a**) LUMO+1 orbital; (**b**) LUMO+2 orbital; **(c)** HOMO-1 orbital; **(d)** HOMO-2 orbital.

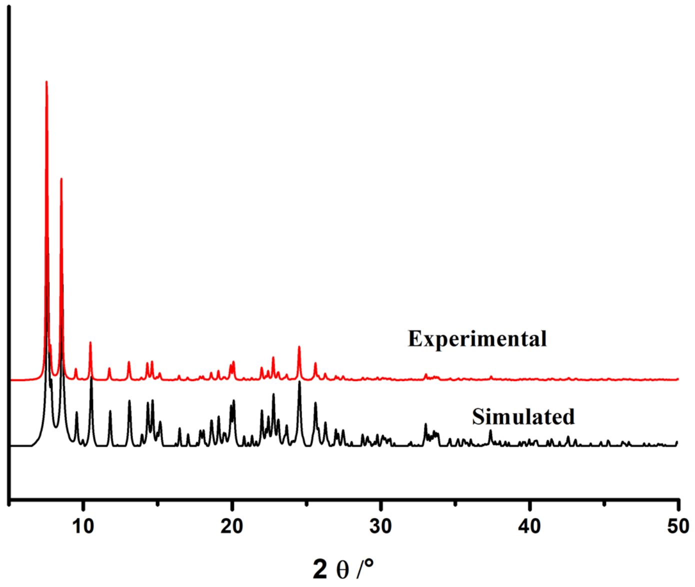

Figure S4. Powder X-ray diffraction (PXRD) patterns of complex **1**

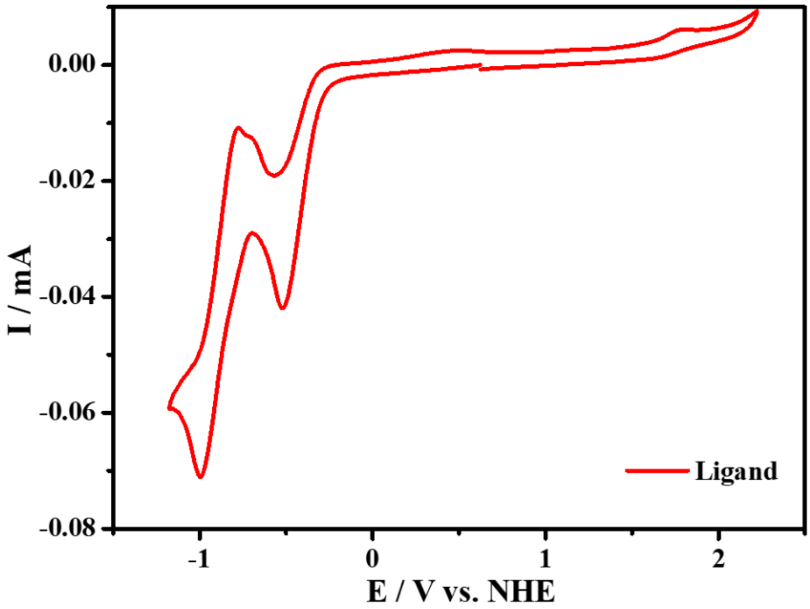

Figure S5. Cyclic voltammetry of 2 mM ligand **L**^{1} under 1 atm Ar at scan rate 100 mV s^{-1}.

Table S5. The reduction potentials and peak currents of complex **1** under 1 atm Ar in a 0.1 M $^{n}Bu_4NPF_6$ CH_3CN supporting electrolyte.

v/V s^{-1}	Potential(V)	Peak current(mA)	Potential(V)	Peak current(mA)	Potential(V)	Peak current(mA)
0.1	-1.38	-0.17	-1.97	-0.44	-2.56	-0.71
0.2	-1.38	-0.29	-1.97	-0.58	-2.62	-0.76
0.3	-1.38	-0.35	-1.97	-0.71	-2.66	-0.79
0.4	-1.38	-0.45	-1.97	-0.91	-2.66	-0.96
0.5	-1.38	-0.51	-1.97	-1.07	-2.66	-1.09

Table S6. The reduction potentials and peak currents of complex **1** under 1 atm CO_2 in a 0.1 M $^nBu_4NPF_6$ CH_3CN supporting electrolyte.

v / V s^{-1}	Potential (V)	Peak current (mA)
0.1	-1.15	-0.09
0.2	-1.22	-0.14
0.3	-1.31	-0.18
0.4	-1.33	-0.24
0.5	-1.35	-0.27

Table S7. The reduction potentials and peak currents of complex **1** containing different concentrations of H_2O under CO_2.

N_{H_2O} (mM)	Potential (V)	Peak current (mA)
0	-1.15	-0.09
0.083	-1.15	-0.115
0.139	-1.15	-0.146

Table S8. The reduction potentials and peak currents of complex **1** with different concentrations under 1 atm CO_2 in a 0.1 M $^nBu_4NPF_6$ CH_3CN supporting electrolyte.

$N_{complex}$ (mM)	Potential (V)	Peak current (mA)
0.5	-1.24	-0.04
1.0	-1.15	-0.065
2.0	-1.15	-0.09
4.0	-1.15	-0.138

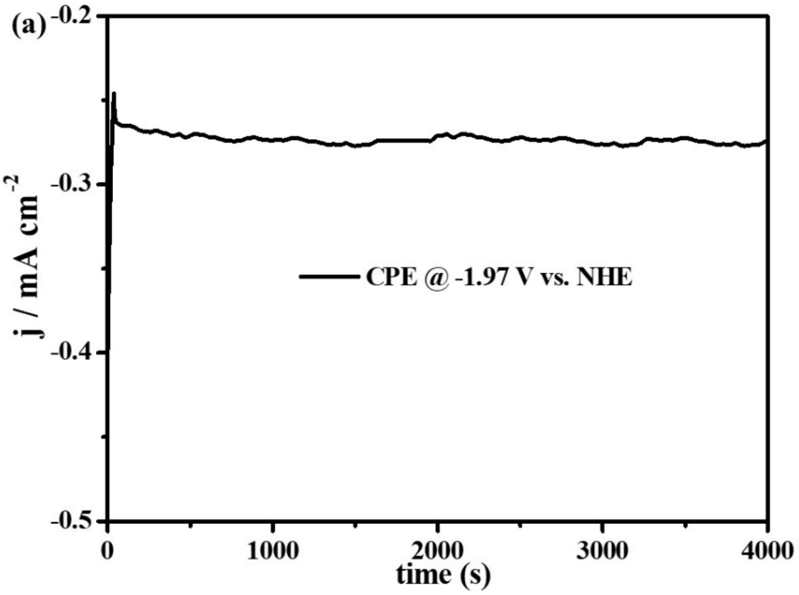

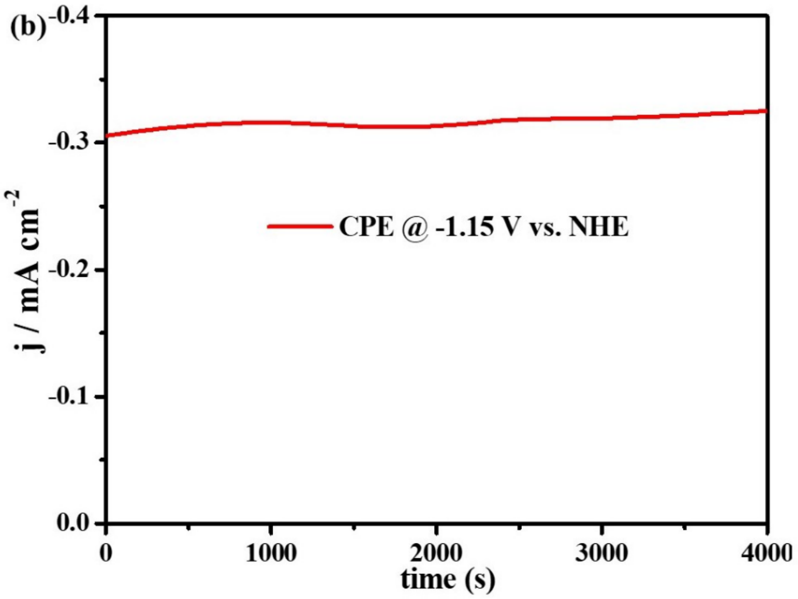

Figure S6. (**a**) CPE for H_2 evolution (black line) at -1.97 V vs NHE on GCE (0.07 cm^2); (**b**) CPE for CO_2 reduction (red line) at -1.15 V vs NHE on GCE (0.07 cm^2) at the same condition as using FTO.

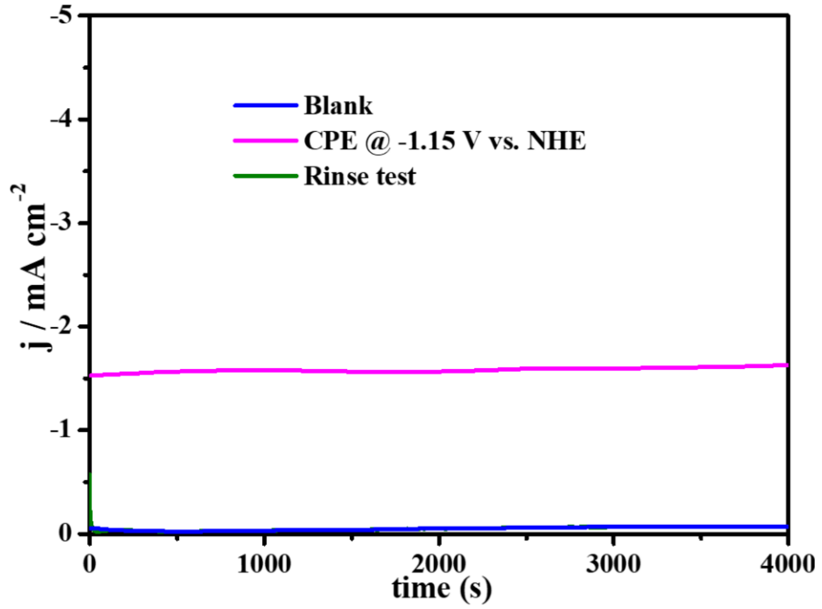

Figure S7. CPE with 2 mM complex **1** (rose red line), rinse test (green line) and the blank experiment without **1** (blue line) on an FTO working electrode (1.0 cm^2).

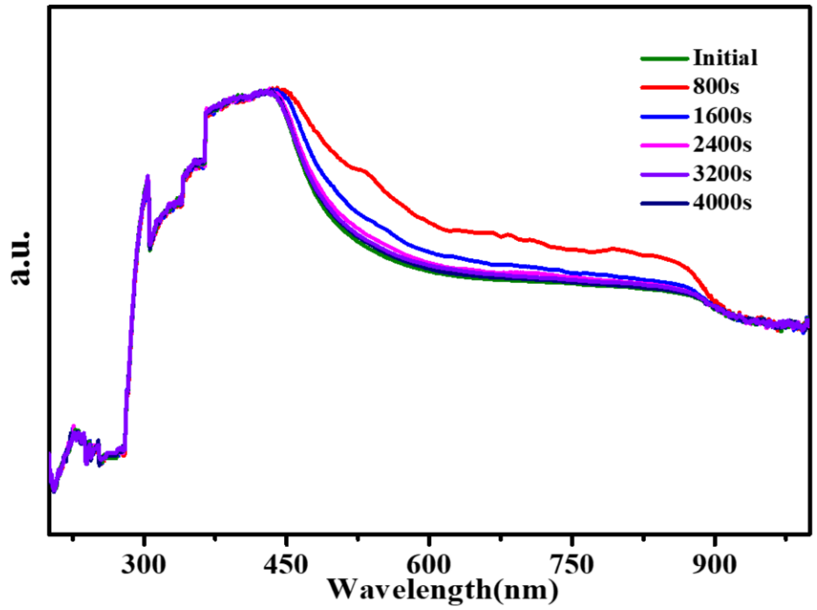

Figure S8. The in-situ UV-Vis spectroelectrochemistry of complex **1** in CO_2 atmosphere.

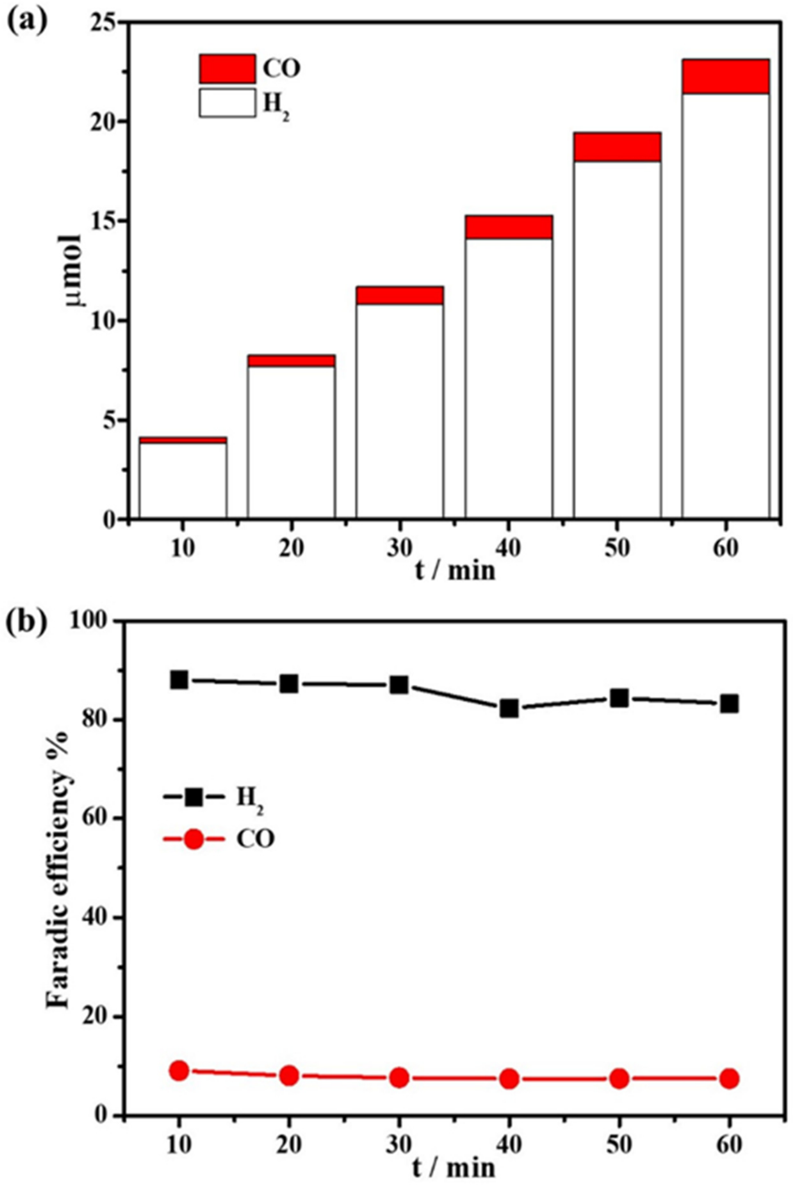

Figure S9. (a) The amount of material in the proton supply system that is combined with the electrocatalytic reduction CO_2 product. **(b)** The Faraday efficiency curves of the electrocatalytic reduction products CO and H_2 in the proton supply H_2O system. Based on the equations (S1) and (S2), the calculated TOF and TON values of the complex **1** for CO_2 reduction are 0.07 s^{-1} and 280, respectively.

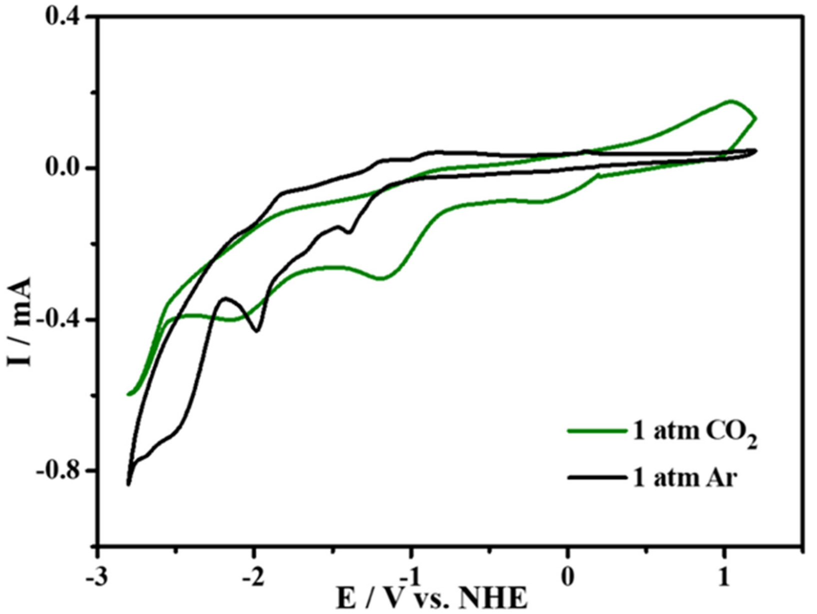

Figure S10. Cyclic voltammetry of complex **1** in the presence (green) and absence (black) of CO_2 recorded at 100 mV s^{-1} at glassy carbon in a 0.1 M $^nBu_4NPF_6$ CH_3CN supporting electrolyte.

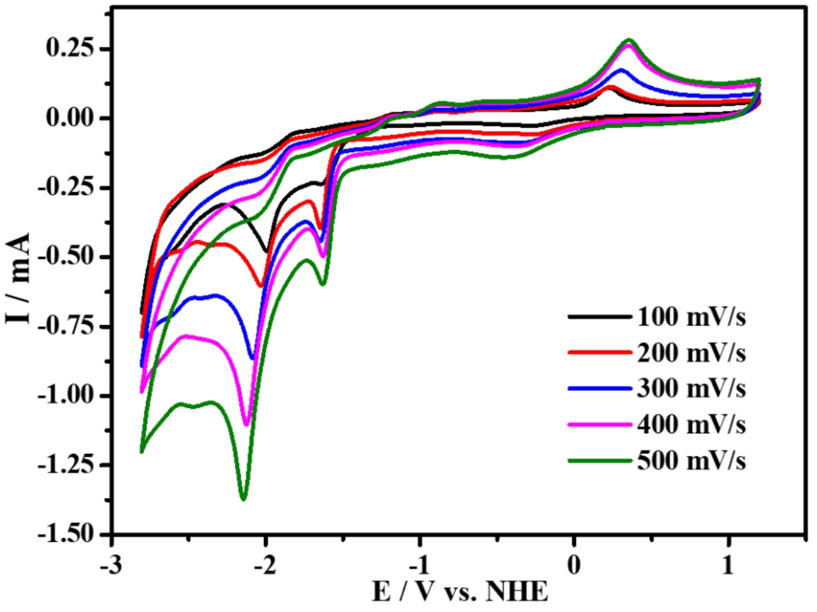

Figure S11. Cyclic voltammograms of complex **1** (2 mM) recorded in the presence of 0.58 mM TsOH·H_2O under 1 atm Ar at scan rate range from 100 to 500 mV s^{-1} in CH_3CN (0.1 M $^nBu_4NBF_6$) at a glassy carbon electrode.

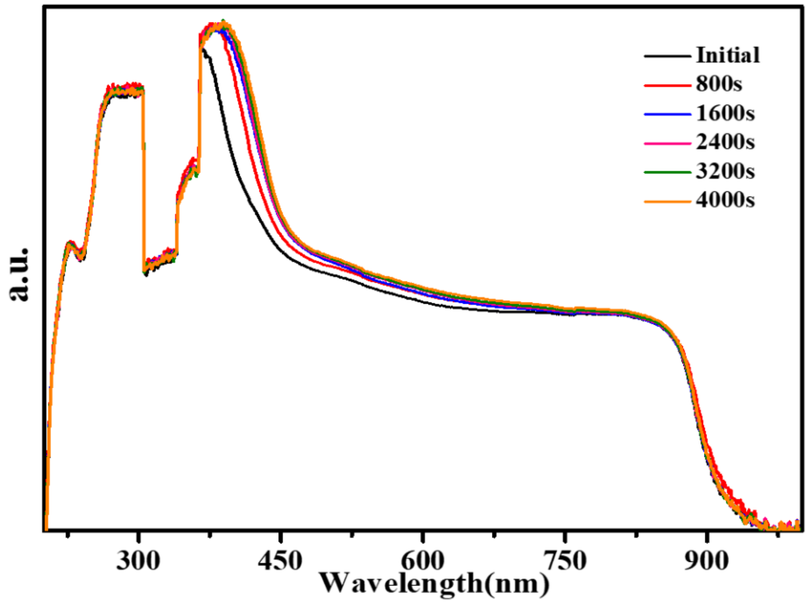

Figure S12. The *in-situ* UV-Vis spectroelectrochemistry of complex **1** in Ar atmosphere.

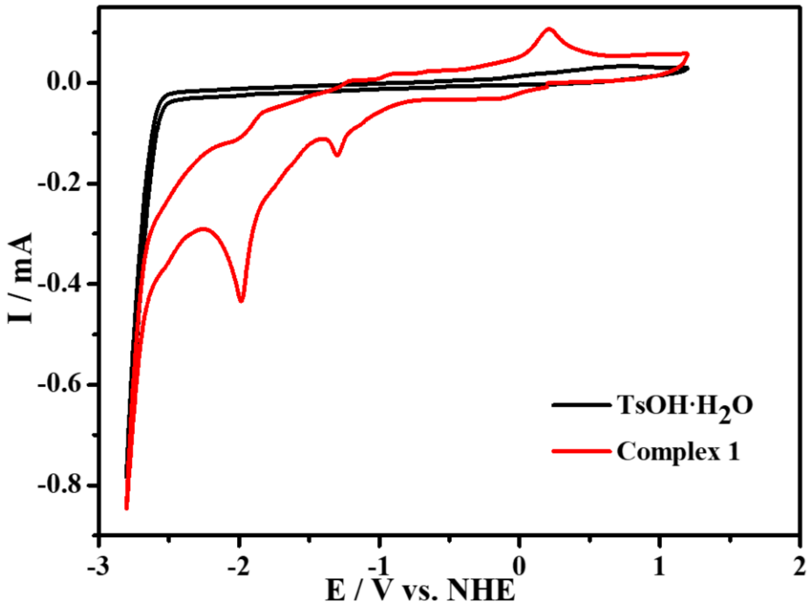

Figure S13. Cyclic voltammograms of complex **1** (2 mM, red trace) and in the presence of 10 mM TsOH·H_2O (black trace) in CH_3CN (0.1 M $^nBu_4NPF_6$) at a glassy carbon electrode and 100 mV s^{-1}.

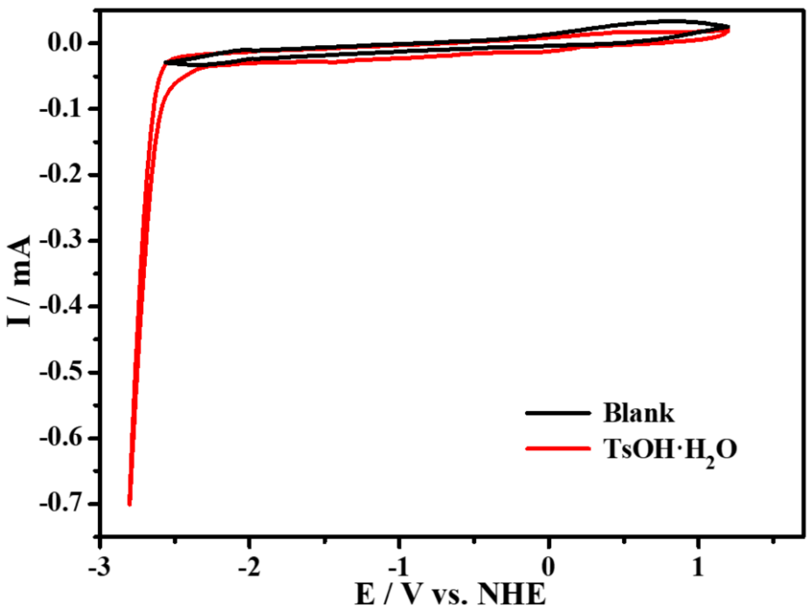

Figure S14. Cyclic voltammograms recorded in the absence (black trace) or in the presence of 10 mM of TsOH·H_2O (red trace). Scan rate: 100 mV s^{-1}. Working electrode: glassy carbon. Counter electrode: Pt wire. Reference electrode: Ag/AgCl.

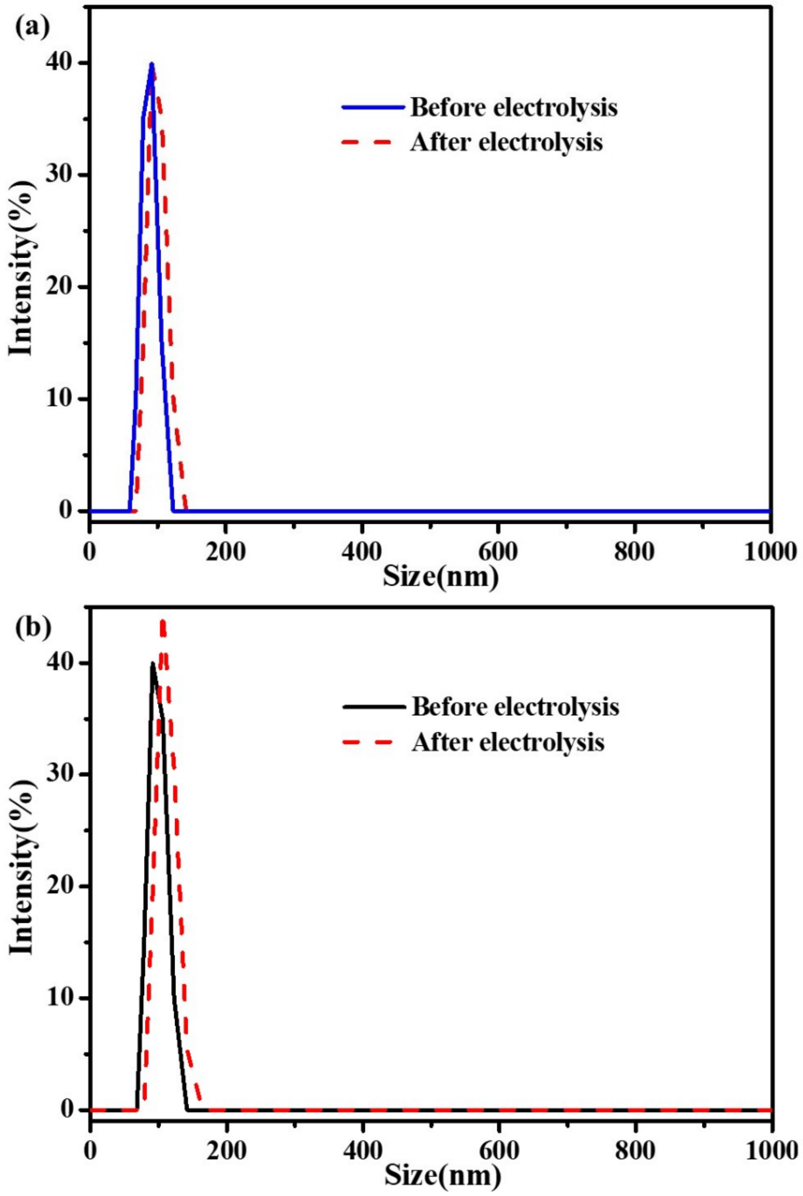

Figure S15. DLS spectra of the electrolyte before and after the CPE test. (**a**) DLS of the complex **1** containing 0.58mM TsOH·H_2O in CH_3CN (0.1 M $^{n}Bu_4NPF_6$) before and after 4000 s electrolysis; (**b**) DLS of the complex **1** in CH_3CN (0.1 M $^{n}Bu_4NPF_6$) under 1atm CO_2 before and after 4000 s electrolysis.

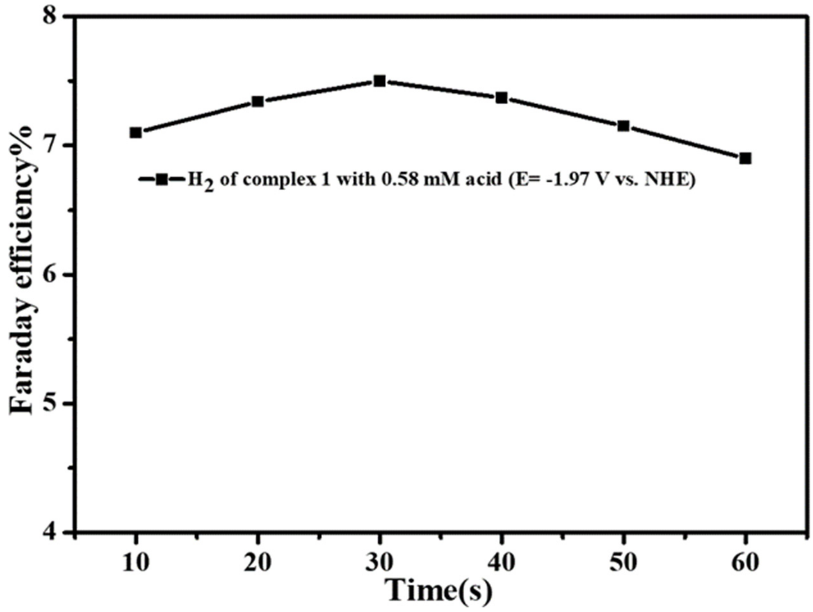

Figure S16. The Faraday efficiency curves of the electrocatalytic hydrogen evolution products H_2 in the proton supply 0.58 mM TsOH·H_2O system.

Controlled Potential Electrolysis: Controlled potential electrolysis takes place in GC-2014C. The FTO is used for the working electrode, the reference electrode is Ag/AgCl electrode containing saturated KCl solution, and the opposite electrode is platinum wire electrode. The working electrolytic contains a 2mM complex, a proton source required for the experiment, and an acetonitrile solution supporting the electrolyte (tetrabutylammonium hexafluorophosphate). The value of TON was calculated according to the electrolytic time of 4000 s. F is the Faraday constant (96485 C·mol^{-1}). n is the number of transferred electrons (n=2).

$$FE=\frac{ppm*V(ml)*n*F}{22.4*Q}100\% \quad (S1)$$

$$Q=it \quad (S2)$$

Based on the equations (S1) and (S2), we have calculated the TOF and TON values of the complex **1** for CO_2 reduction, which are 0.07s^{-1} and 280, respectively, as shown in the caption of Figure S9.

During hydrogen evolution test, after adding 0.58 mM of p-toluenesulfonic acid, FE of H_2 is about 7.6% and TON = 78, as shown in Figure S16.

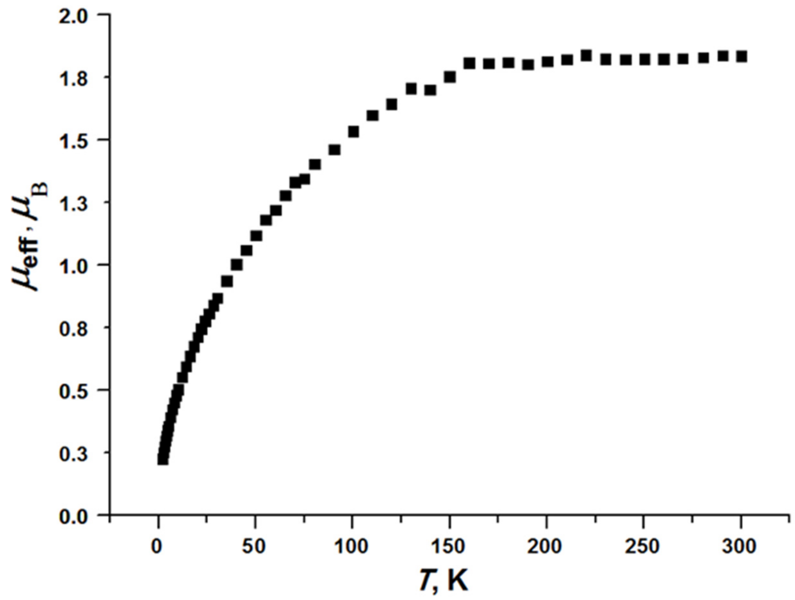

Figure S17. Plot of the magnetic moments (μ_{eff}) versus the temperature T for solid sample of complex **1**.

Table S9. The molecular geometry of complex **1** as predicted by SHAPE 2.1.

S H A P E v2.1		Continuous Shape Measures calculation	
(c) 2013 Electronic Structure Group, Universitat de Barcelona			
vOC-2		3 C2v	Tetravacant octahedron
L-2		1	Dinfh Linear

Structure [ML2]		vOC-2	L-2
DOSDAQ	1 Cu,	52.336,	92.312
O	1 O ,	11.761,	22.868
O	0 O ,	27.345,	29.899
N	4 N ,	36.365,	74.167
N	9 N ,	0.410,	27.040
